# Hp-s1 Ganglioside Suppresses Proinflammatory Responses by Inhibiting MyD88-Dependent NF-κB and JNK/p38 MAPK Pathways in Lipopolysaccharide-Stimulated Microglial Cells

**DOI:** 10.3390/md18100496

**Published:** 2020-09-29

**Authors:** Jui-Hu Shih, Yow-Fu Tsai, I-Hsun Li, Ming-Hua Chen, Yuahn-Sieh Huang

**Affiliations:** 1 Department of Pharmacy Practice, Tri-Service General Hospital, Taipei 11490, Taiwan; jtlovehl@gmail.com (J.-H.S.); lhs01077@gmail.com (I-H.L.); 2 School of Pharmacy, National Defense Medical Center, Taipei 11490, Taiwan; 3 Department of Chemistry, Chung Yuan Christian University, Taoyuan 32023, Taiwan; tsaiyofu@cycu.edu.tw; 4 Division of Neurology, Department of Medicine, Armed Forces Taoyuan General Hospital, Taoyuan 32551, Taiwan; minghua1833@gmail.com; 5 Department of Neurology, Tri-Service General Hospital, National Defense Medical Center, Taipei 11490, Taiwan; 6 Department of Biology and Anatomy, National Defense Medical Center, Taipei 11490, Taiwan

**Keywords:** ganglioside Hp-s1, microglia, lipopolysaccharide, neuroinflammation

## Abstract

Hp-s1 ganglioside is isolated from the sperm of sea urchin (Hemicentrotus pulcherrimus). In addition to neuritogenic activity, the biological function of Hp-s1 in neuroinflammation is unknown. In this study, we investigated the anti-neuroinflammatory effect of Hp-s1 on lipopolysaccharide (LPS)-stimulated microglial cells. MG6 microglial cells were stimulated with LPS in the presence or absence of different Hp-s1 concentrations. The anti-inflammatory effect and underlying mechanism of Hp-s1 in LPS-activated microglia cells were assessed through a Cell Counting kit-8 assay, Western blot analysis, and immunofluorescence. We found that Hp-s1 suppressed not only the expression of inducible nitric oxide synthase and cyclooxygenase-2 but also the expression of proinflammatory cytokines, such as TNF-α, IL-1β, and IL-6. Hp-s1 inhibited the LPS-induced NF-κB signaling pathway by attenuating the phosphorylation and translocation of NF-κB p65 and by disrupting the degradation and phosphorylation of inhibitor κB-α (IκBα). Moreover, Hp-s1 inhibited the LPS-induced phosphorylation of p38 mitogen-activated protein kinase (MAPK) and c-Jun *N*-terminal kinase (JNK). Hp-s1 also reduced the expression of myeloid differentiation factor 88 (MyD88) and TNF receptor-associated factors 6 (TRAF6), which are prerequisites for NF-κB and MAPKs activation. These findings indicated that Hp-s1 alleviated LPS-induced proinflammatory responses in microglial cells by downregulating MyD88-mediated NF-κB and JNK/p38 MAPK signaling pathways, suggesting further evaluation as a new anti-neuroinflammatory drug.

## 1. Introduction

Microglia are resident macrophages in the brain, and they participate in the innate immune defense and neuroinflammatory responses of the central nervous system [1]. Activated microglia can release proinflammatory mediators, including tumor necrosis factor-α (TNF-α), interleukin-1β (IL-1β), IL-6, nitric oxide (NO), and prostaglandin E2 (PGE2). The overactivation or sustained activation of microglia can cause severe neurotoxicity by producing excessive proinflammatory mediators [2,3]. Extensive evidence has demonstrated that controlling microglial activation can alleviate the severity of neurodegenerative diseases [3,4]. 

Lipopolysaccharide (LPS) is the main component of the outer membrane of Gram-negative bacteria and acts as a potent stimuli for microglial activation [5]. LPS-stimulated microglial cells are common and useful models for studying the mechanisms of neuroinflammation and neurological disorders [5]. LPS can bind to and activate toll-like receptor 4 (TLR4), leading to activation of nuclear factor κB (NF-κB) and mitogen-activated protein kinases (MAPKs) [6]. Previous studies showed that NF-κB and MAPKs play key roles in the production of proinflammatory mediators and enzymes, such as TNF-α, IL-1β, IL-6, inducible nitric oxide synthase (iNOS), and cyclooxygenase-2 (COX-2) [7,8].

Gangliosides are sialic acid-containing glycosphingolipids that are components of mammalian cell membranes [9]. Emerging evidence has suggested that gangliosides are not passive structural components of cell membranes; instead, they are modulators of important biological processes, such as cell-cell recognition, adhesion, differentiation, neural plasticity, and inflammation [10]. Gangliosides are particularly abundant in the nervous system and implicated in the physiology and pathology of the brain [10,11]. Numerous studies have reported that the administration of certain gangliosides has shown neurotrophic and neuroprotective potential in vitro and in experimental animal models of neurological disorders, such as stroke, spinal cord injury, Parkinson’s disease, and Alzheimer’s disease [10,11,12]. In inflammatory regulation, the role of gangliosides in neuroinflammation remains controversial. Nikolaeva et al. (2015) showed that GM1 and GD1a gangliosides reduce LPS-induced signaling in PC12 pheochromocytoma cells [13]. They found that these gangliosides minimize the translocation of TLR4 into lipid rafts required for TLR4 signaling. However, some gangliosides can induce the expression of COX-2 and the production of NO and TNF-α in rat microglial cells [14]. Jou et al. (2006) reported that gangliosides also elicit proinflammatory effects that may involve TLR4 activation [15]. Neurodegeneration in mice is caused by the genetic depletion of glycosyltransferase genes responsible for ganglioside synthesis [16]. Astrocyte proliferation, microglial infiltration, and inflammatory cytokine production are upregulated in knockout mice compared with those in wild-type mice; thus, they contribute to the onset of neurological disorders in mice [17,18,19].

Hp-s1 ganglioside is isolated from the sperm of Hemicentrotus pulcherrimus or the ovary of Diadema setosum [20]. Studies on gangliosides extracted from marine invertebrates have demonstrated neuritogenic activities in the rat pheochromocytoma cell line PC-12 in the presence of nerve growth factor (NGF) [21]. The neuritogenic activity of Hp-s1 and its synthetic analogs is better than that of GM1 ganglioside in PC-12 cells [22]. Moreover, Hp-s1 analog inhibits amyloidogenic toxicity in model cells of Alzheimer’s disease [23]. However, the effects of Hp-s1 on neuroinflammation are still unknown. 

This study demonstrated the anti-neuroinflammatory effect of Hp-s1 on LPS-stimulated microglia. Our results suggested that Hp-s1 might be a drug candidate for the treatment of inflammation-related neurological diseases.

## 2. Results

### 2.1. Cell Viability Is Not Affected by Hp-s1 Ganglioside

**Figure 1:**
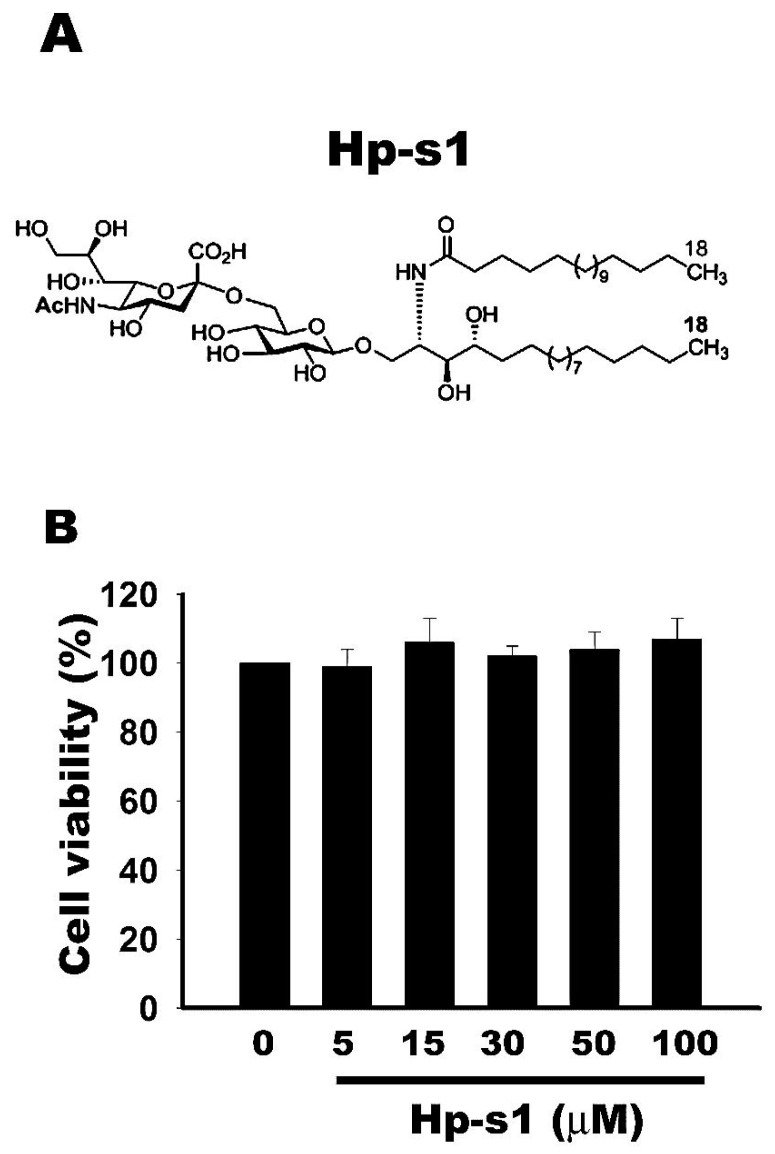
Effect of Hp-s1 ganglioside on cell viability of MG6 microglial cells. The cells were cultured with different Hp-s1 concentrations (5–100 μM) for 24 h. The cell viability was determined via a CCK-8 assay. Quantitative data were expressed as the percentage of the corresponding untreated control value (mean ± S.D., *n* = 3, quadruplicate wells for each condition).

MG6 microglial cells were treated with different Hp-s1 concentrations ranging from 0 μM to 100 μM to investigate the cytotoxic effect of Hp-s1. After 24 h, cell viability was determined via the Cell Counting kit-8 (CCK-8) assay. As shown in Figure 1, the results showed that cell viabilities were not affected by Hp-s1 at concentrations of up to 100 μM. Hence, Hp-s1 exerted no significant cytotoxic effect on MG6 microglial cells.

### 2.2. Hp-s1 Inhibits the LPS-Induced Expression of iNOS and COX-2

**Figure 2:**
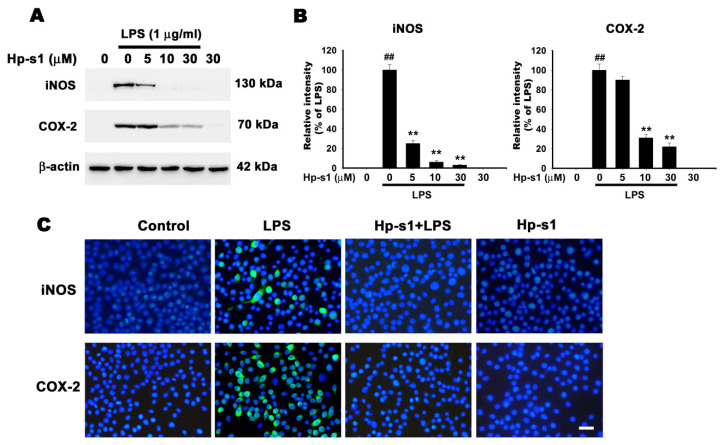
Hp-s1 suppresses the lipopolysaccharide (LPS)-induced expression of iNOS and COX-2 in a dose-dependent manner. (**A**) The cells were pretreated with Hp-s1 (5, 15, or 30 μM) for 2 h and then treated with or without LPS (1 μg/mL) for another 8 h. Total lysates were collected, and the protein levels of iNOS and COX-2 were detected via Western blot analysis. β-actin was used as an internal control. (**B**) The protein bands of each regimen were quantified through densitometry. Data were expressed as the percentage of the LPS-treated group (mean ± S.D., *n* = 3). ^##^ *p* < 0.01 vs. the control group; ** *p* < 0.01 vs. the LPS-treated group. (**C**) Immunofluorescence staining for iNOS and COX-2. Cells were pretreated with 30 μM Hp-s1 for 2 h and then stimulated with or without LPS (1 μg/mL) for 8 h. The cells were immunostained with anti-iNOS and anti-COX-2 antibodies. The nuclei (blue) were stained with 4′,6-diamidino-2-phenylindole (DAPI). Bar = 30 μm.

iNOS and COX-2 are predominant proinflammatory enzymes that participate in the synthesis of NO and PGE2, respectively. Thus, we examined the effect of Hp-s1 on the protein levels of iNOS and COX-2 via Western blot. The results showed that Hp-s1 attenuated the LPS-stimulated protein expression of iNOS and COX-2 in a dose-dependent manner, whereas Hp-s1 alone did not affect their protein expression (Figure 2A,B). It is noteworthy that the inhibitory effect of Hp-s1 on iNOS is larger than COX-2. Immunofluorescence staining with anti-iNOS and anti-COX-2 antibodies also confirmed that Hp-s1 suppressed the expression of iNOS and COX-2 in LPS-stimulated microglial cells (Figure 2C).

### 2.3. Hp-s1 Suppresses the LPS-Induced Expression of Proinflammatory Cytokines in Microglial Cells 

**Figure 3:**
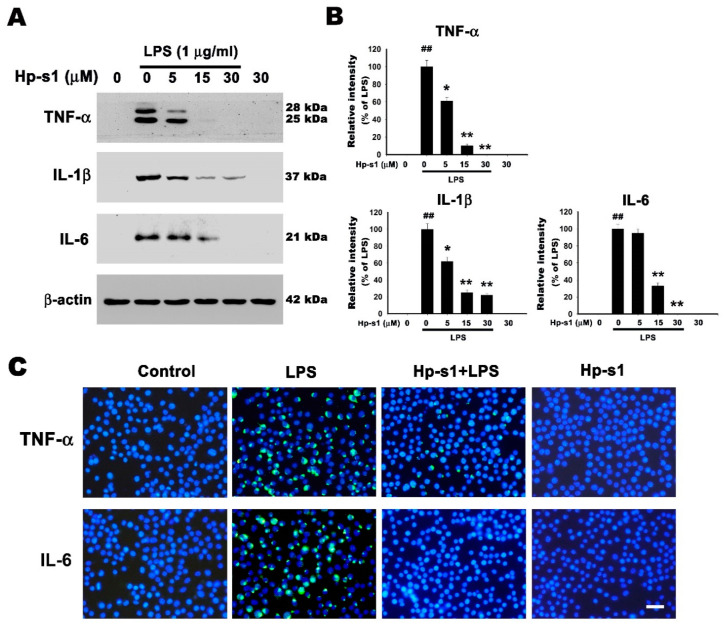
Hp-s1 suppresses proinflammatory cytokines in LPS-stimulated microglial cells. (**A**) The cells were pretreated with different Hp-s1 concentrations (5, 15, or 30 μM) in the absence or presence of LPS (1 μg/mL) for 8 h and then subjected to Western blot analysis with anti-TNF-α, anti-IL-1β, and IL-6 antibodies; β-actin was used as an internal control. (**B**) The protein bands of each regimen were quantified via densitometry. Data were expressed as the percentage of the LPS-treated group (mean ± S.D., *n* = 3). ^##^ *p* < 0.01 vs. the control group; * *p* < 0.05 and ** *p* < 0.01 vs. the LPS-treated group. (**C**) Immunofluorescence staining for TNF-α. and IL-6. The cells were pretreated with 30 μM Hp-s1 for 2 h and then stimulated with or without LPS (1 μg/mL) for 8 h. The cells were immunostained with anti-TNF-α and IL-6 antibodies. The nuclei (blue) were stained with DAPI. Bar = 30 μm.

The production of proinflammatory cytokines is a critical feature of LPS-activated microglial cells [24]. Hence, the effects of Hp-s1 on the LPS-induced production of proinflammatory mediators were examined through Western blot analysis. In Figure 3A,B, the LPS administration significantly increased the production of proinflammatory cytokines, including TNF-α, IL-1β, and IL-6; conversely, the pretreatment with Hp-s1 decreased the production of TNF-α, IL-1β, and IL-6 in a dose-dependent manner. The treatment of Hp-s1 alone had no effects on the production of these proinflammatory factors compared with that in the control group (Figure 3A,B). Immunofluorescence staining with anti-TNF-α and anti-IL-6 also confirmed that Hp-s1 suppressed the expression of TNF-α and IL-6 in LPS-treated microglial cells (Figure 3C). These results indicated that Hp-s1 exerted an anti-proinflammatory response on LPS-stimulated MG6 cells.

### 2.4. Hp-s1 Inhibits the LPS-Induced NF-κB Activation

**Figure 4:**
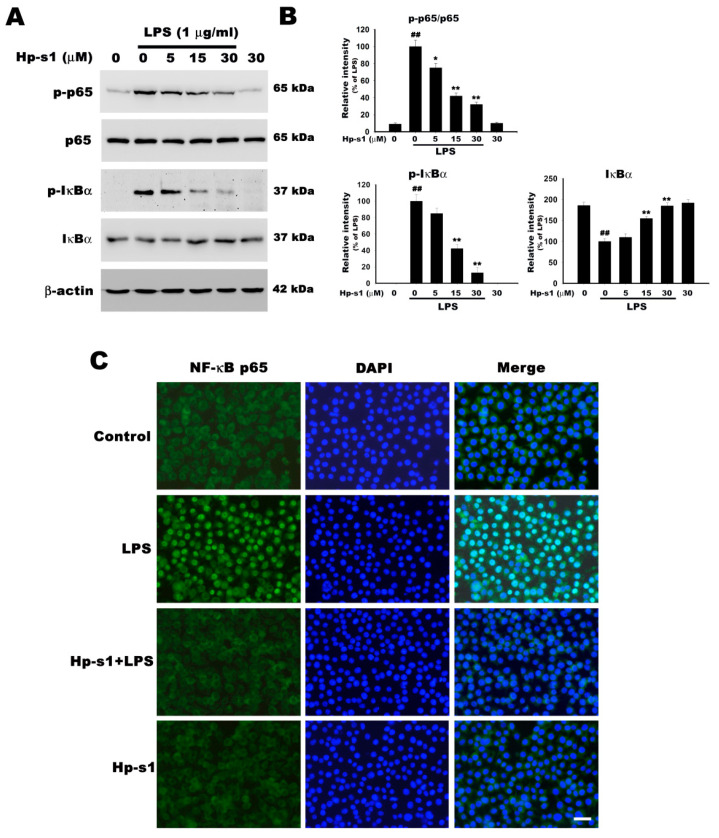
Hp-s1 inhibits the LPS-induced NF-κB activation. (**A**) The cells were pretreated with different Hp-s1 concentrations (5, 15, or 30 μM) for 2 h and then stimulated with LPS (1 μg/mL) for 1 h. Cell lysates were subjected to Western blot to determine the protein levels of p-p65, p65, p-IκBα, and IκBα. β-actin was used as an internal control. (**B**) The protein bands of each regimen were quantified via densitometry. Data were expressed as the percentage of the LPS-treated group (mean ± S.D., *n* = 3). ^##^ *p* < 0.01 vs. the control group; * *p* < 0.05 and ** *p* < 0.01 vs. the LPS-treated group. (**C**) Immunofluorescence analysis of NF-κB translocation. The cells were pretreated with Hp-s1 (30 μM) for 2 h and then activated with LPS (1 μg/mL) for 1 h. The translocation of the p65 subunit of NF-κB was determined via immunocytochemistry using anti-p65 NF-κB antibody. The nuclei (blue) were stained with DAPI. Scale bar = 30 μm.

The activation and phosphorylation of NF-κB play an essential role in the production of iNOS, COX-2, and proinflammatory cytokines [1]. The effects of Hp-s1 on LPS-induced NF-κB activation were evaluated in terms of the tyrosine phosphorylation of p65 and IκB, and the degradation of IκB to further elucidate the mechanisms of Hp-s1 on the LPS-stimulated microglial activation. As shown in Figure 4A,B, the phosphorylation of p65 markedly increased in the LPS-stimulated microglial cells. Hp-s1 pretreatment significantly suppressed the LPS-induced increase in p-p65 in a concentration-dependent manner. In addition, the LPS administration promoted the IκBα degradation through the phosphorylation of IκBα, and this increased degradation was significantly attenuated by Hp-s1 through attenuating IκBα phosphorylation in a concentration-dependent manner. The treatment of Hp-s1 alone had no effects on the NF-κB activation (Figure 4A,B). The inhibitory effect of Hp-s1 on the nuclear translocation of NF-κB was further confirmed by immunofluorescence. As shown in Figure 4C, in control cells, NF-κB p65 was mostly localized in the cytoplasm. In contrast, immunofluorescence staining of NF-κB p65 was increased in the nucleus of LPS-stimulated cells within 1 h. Hp-s1 treatment markedly inhibited the LPS-induced nuclear translocation of NF-κB. Hp-s1 alone did not affect the cytoplasmic distribution (Figure 4C). Therefore, we inferred that Hp-s1 inhibited proinflammatory enzymes and cytokines by inhibiting the translocation and activity of NF-κB p65 activity.

### 2.5. Hp-s1 Inhibits the LPS-Induced Activation of JNK and p38 MAPK

**Figure 5:**
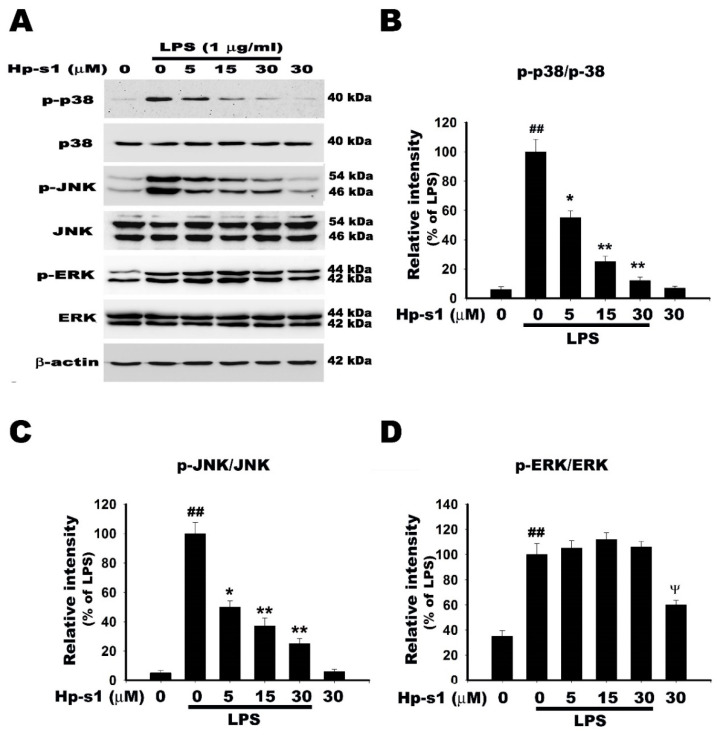
Hp-s1 inhibits the LPS-induced JNK/p38 MAPK activation. (**A**) The cells were pretreated with different Hp-s1 concentrations (5, 15, or 30 μM) for 2 h and then stimulated with or without LPS (1 μg/mL) for 1 h. Cell lysates were subjected to Western blot to determine the protein levels of MAPKs (p-p38/p38, p-JNK/JNK, and p-ERK/ERK). β-actin was used as an internal control. (**B**–**D**) The protein bands of each regimen were quantified via densitometry. Data were expressed as the percentage of the LPS-treated group (mean ± S.D., *n* = 3). ^##^ *p* < 0.01 and ^ᴪ^ *p* < 0.05 vs. the control group; * *p* < 0.05 and ** *p* < 0.01 vs. the LPS-treated group.

Each of the three MAPK pathways, namely, p38 MAPK, c-Jun *N*-terminal kinase (JNK), and extracellular signal-related kinases (ERK), is implicated in the regulation of NF-κb activation and the production of proinflammatory mediators [7,25]. Therefore, the effect of Hp-s1 on the LPS-induced MAPK activation was assessed in terms of the phosphorylation of p38 MAPK, JNK, and ERK through Western blot analysis. As shown in Figure 5, the stimulation of MG6 cells with LPS resulted in the phosphorylation of p38, JNK, and ERK. Hp-s1 attenuated the LPS-induced phosphorylation of p38 MAPK and JNK in a concentration-dependent manner, but left ERK unaffected. The treatment with Hp-s1 alone had no effects on the phosphorylation of the p38 and JNK, whereas it increased in phosphorylation of ERK compared with control group (Figure 5A,D). These results suggest that regulation of JNK and p38 MAPK signaling cascade is another possible mechanism underlying inhibitory effect of Hp-s1 in LPS-stimulated microglia.

### 2.6. Hp-s1 Blocks the LPS-Induced MyD88/TRAF6-Dependent Signaling Pathway

**Figure 6:**
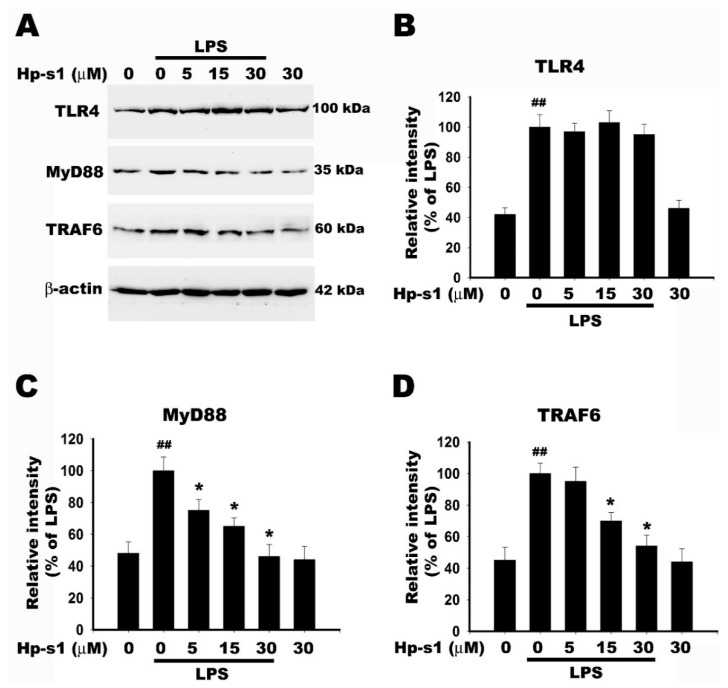
Hp-s1 blocks the LPS-induced MyD88/TRAF6-dependent signaling pathway in MG6 microglial cells. (**A**) The cells were pretreated with different Hp-s1 concentrations (5, 15, or 30 μM) for 2 h and then stimulated with or without LPS (1 μg/mL) for 40 min. Cell lysates were subjected to Western blot to determine the protein levels of TLR4, MyD88, and TRAF6. β-actin was used as an internal control. (**B**–**D**) The protein bands of each regimen were quantified via densitometry. Data were expressed as the percentage of the LPS-treated group (mean ± S.D., *n* = 3). ^##^ *p* < 0.01 vs. the control group; * *p* < 0.05 vs. the LPS-treated group.

LPS-induced TLR4 activation triggers two pathways, namely, MyD88-dependent and MyD88-independent pathways. The MyD88-dependent pathway is initiated by the recruitment of MyD88 to the cytosolic Toll/interleukin-1 receptor (TIR) domain of TLR4, and IRAK-4 and IRAK-1 (IL-1R-associated kinases) are consequently activated; as a result, IRAK1 can recruit TNF receptor-associated factor-6 (TRAF6). The IRAK1–TRAF6 complex phosphorylates TAK1 and TAB-1, a related binding protein, leading to the activation of NF-κB, p38 MAPK, and JNK signaling pathways [25,26,27]. We examined whether Hp-s1 affected the LPS-induced TLR4/MyD88/TRAF6-dependent pathway. As shown in Figure 6, LPS alone induced an increase in the protein expression of TLR4, MyD88, and TRAF6. Hp-s1 pretreatment reduced the LPS-induced the upregulation of protein level of MyD88 and TRAF6, but left TLR4 unaffected. An amount of 30 μM Hp-s1 alone did not affect the expression of TLR4, MyD88, and TRAF6 compared to the control group. These results suggested that Hp-s1 mitigated the LPS-induced proinflammatory response through the MyD88/TRAF6-mediated signaling pathway.

## 3. Discussion

Hp-s1 ganglioside is one of the ingredients isolated from the sperm of sea urchin (Hemicentrotus pulcherrimus) [20]. In our study, we first demonstrated that the exogenous Hp-s1 administration could attenuate LPS-stimulated microglial activation, and that its inhibitory effect was not mediated by cytotoxicity. In addition, our results indicated that Hp-s1 could inhibit the production of proinflammatory mediators or enzymes by suppressing the MyD88/TRAF6-dependent NF-κb and JNK/p38 MAPK signaling pathways, which might be one of the mechanisms responsible for the anti-neuroinflammatory effect of Hp-s1.

LPS is one of the most effective stimulants that activate microglia. Microglial activation produces increased levels of proinflammatory enzymes or mediators, including iNOS, COX2, TNF-α, IL-6, and IL-1β. These inflammatory mediators participate in neuroinflammation and cause various neurological disorders. Hence, suppressing the activation of microglia and the production of proinflammatory cytokines can contribute to the development of effective treatments for neurodegenerative diseases [26]. iNOS and COX-2 are proinflammatory enzymes that generate NO and PGE2, respectively. Increased NO and PGE2 levels are observed in the cerebrospinal fluid of patients with neuroinflammatory diseases. The proinflammatory cytokines TNF-α, IL-1β, and IL-6 are also implicated in the pathogenesis of neuroinflammatory diseases [28]. In this study, our results indicated that Hp-s1 significantly inhibited iNOS, COX-2 TNF-α, IL-6, and IL-1β production in LPS-stimulated MG6 microglial cells. These results revealed that Hp-s1 elicited anti-inflammatory effects on LPS-activated MG6 microglial cells.

This study examined whether Hp-s1 affected the activation of NF-κB, which is an important inducible transcription factor required to control DNA transcription and cytokine production in response to inflammation, to further clarify the anti-inflammatory mechanism mediated by Hp-s1. NF-κB is a protein complex whose subunits are capable of homo- or heterodimerizing to form transcription factors, thereby regulating the transcription of target genes. The most abundant form of activated NF-κB is a heterodimer of p50 and p65, which contains transcriptional activation domains necessary for gene production. Before activation, NF-κB exists as a heterodimer in the cytoplasm by physically binding to IκBα. After LPS stimulation, IκBα is phosphorylated by IκB kinase, dissociated by NF-κB, and subsequently degraded via the ubiquitin–proteasome pathway. The free NF-κB dimers are converted into active forms, phosphorylated at p65, and then translocated to the nucleus, where they act as a transcription factor of proinflammatory genes [1,29]. In the present study, Hp-s1 strongly inhibited the LPS-stimulated NF-κB p65 phosphorylation, translocation, and activation by suppressing the phosphorylation and degradation of IκBα. These results indicated that Hp-s1 suppressed the LPS-induced upregulation of proinflammatory enzymes and cytokines by inhibiting the activation of NF-κB p65.

MAPKs, which are a family of serine/threonine protein kinases, mediate various cell processes in response to external stress signals [30,31]. Their activation is involved in the production of inflammation mediators; as such, they are potential therapeutic targets for anti-inflammatory responses [7]. Activated MAPKs phosphorylate various substrate proteins, including proinflammatory transcription factors, such as c-Jun, c-Fos, Elk-1, activating transcription factor 2 (ATF2), and myocyte enhancer factor; thus, they positively regulate gene transcription [32,33]. In microglia, TLR4 signaling stimulated by LPS increases the phosphorylation of MAPKs, including JNK, p38 MAPK, and ERK, and upregulates the expression of proinflammatory cytokines, chemokines, and inflammatory responses [34]. In addition, MAPKs participate in the positive regulation of the NF-κB transcriptional activity [35]. In our results, Hp-s1 attenuated the LPS-induced phosphorylation of JNK and p38 MAPK in a dose-dependent manner compared with LPS-treated cells, whereas p-ERK was not affected following treatment of the compound. It is worth noting that Hp-s1 alone increased the phosphorylation of ERK in microglial cells. Several previous studies reported that some gangliosides increase the phosphorylation of ERK, such as GM1 [36], Hp-s1 analog [23], and GM3 [37]. Increase in phosphorylation of ERK by Hp-s1 might explain why Hp-s1 cannot inhibit ERK activity in LPS-stimulated microglia. However, the detailed mechanism requires further investigation. In summary, these results indicate that the inhibition of JNK/p38 MAPK activation was implicated in the anti-proinflammatory response of Hp-s1 in LPS-stimulated microglial cells.

TLR4 signaling of LPS stimulation requires for induction of cytokines and other inflammatory mediators in microglia [38,39]. Upon LPS stimulation, TLR4 induces oligomerization and recruits downstream adaptor proteins through its interaction with the cytosolic TIR domains of TLR4. Five TIR domain-containing adaptor proteins (MyD88, TIRAP, TRIF, TRAM, and SARM) have been identified, and they are essential for signaling transduction [40]. TLR4/MyD88-dependent signaling recruits IRAK1/TRAF6 complex, leading to TAK1 phosphorylation and subsequently activates NF-κB and MAPKs by phosphorylating IκBα and MAPK signaling pathways [39,41]. TLR4, MyD88, and TRAF6 are weakly expressed by resting cells, but they upregulate following stimulation with LPS [42]. In our study, although Hp-s1 pretreatment did not affect TLR4 expression, Hp-s1 attenuated LPS-induced the increase in the expression of MyD88 and TRAF6, which contributes to suppressing proinflammatory responses. Overall, the Hp-s1 attenuated NF-κB and JNK/p38 MAPK activities by blocking MyD88/TRAF6-dependent signaling in the LPS-stimulated microglial cells.

Previous studies indicated that several gangliosides, such as GM3 and GD1a, trigger anti-inflammatory responses in LPS-stimulated RAW264.7 macrophages [43,44]. In the present study, we provided evidence that Hp-s1 had an anti-neuroinflammatory role in LPS-stimulated microglial cells. Nonsteroidal anti-inflammatory drugs (NSAIDs), such as naproxen, ibuprofen, and aspirin, are widely administered as anti-inflammatory prescription drugs, but their long-term use may cause stomach ulcers, kidney damage, stroke, or heart attacks [45]. Corticosteroids are a class of steroid hormones that prevent inflammatory conditions [46], but they also induce side effects, including Cushing’s syndrome, hypertension, hyperglycemia, osteoporosis, and connective tissue weakness [46,47]. Marine drugs are increasingly used as agents or supplements to control inflammation because of their efficacy and safety, and studies are being conducted to identify novel natural drugs. However, there are still some limitations in this study, such as whether Hp-s1 crosses the blood-brain barrier, or whether it affects the interaction between LPS and TLR4. The detailed mechanisms in primary microglia and animal model of neuroinflammation require further investigation.

**Figure 7:**
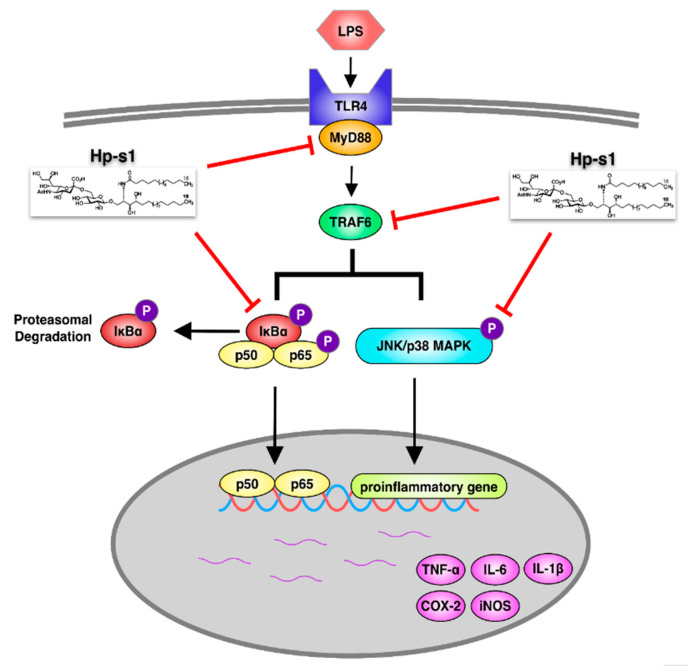
Schematic of the anti-inflammatory action of Hp-s1. Hp-s1 inhibited the proinflammatory reactions in MG6 microglial cells by repressing the MyD88/TRAF6-dependent NF-κB and JNK/p38 MAPK signaling pathways.

In summary, our investigation indicated that Hp-s1 elicited a potential inhibitory effect on the LPS-stimulated proinflammatory mediator production by suppressing the MyD88/TRAF6-mediated activation of NF-κB and p38 MAPK/JNK signaling (Figure 7). Therefore, these observations suggested that Hp-s1 ganglioside exhibits potential for application in preventing a variety of neuroinflammatory diseases by suppressing microglial activation.

## 4. Materials and Methods

### 4.1. Antibodies

The following antibodies were used in this study: rabbit anti-iNOS (Catalog number: sc-650, Santa Cruz Biotechnology, Santa Cruz, CA), rabbit anti-COX-2 (Catalog number: 12282, Cell Signaling, Danvers, MA, USA), rabbit anti-TNF-α (Catalog number: 11948, Cell Signaling), mouse anti-IL-1β (Catalog number: 12242, Cell Signaling), rabbit anti-IL-6 (Catalog number: 12912, Cell Signaling), phospho-NF-κb p65 (Ser536; Catalog number: 3033, Cell Signaling, Danvers, MA, USA), rabbit anti-NF-κb p65 (Catalog number: 3034, Cell Signaling), rabbit anti-p-IκB (Ser32/36; Catalog number: 9246, Cell Signaling), mouse anti-IκB (Catalog number: 4814, Cell Signaling), rabbit anti-p-p38 MAPK (Thr180/Tyr182; Catalog number: 9215, Cell Signaling), rabbit anti-p38 MAPK (Catalog number: 9212, Cell Signaling), rabbit anti-p-JNK (Thr183/Tyr185; Catalog number: 4668, Cell Signaling), rabbit anti-JNK (Catalog number: 9252, Cell Signaling), rabbit anti-p-ERK (Thr202/Tyr204; Catalog number: 4377, Cell Signaling), rabbit anti-ERK (Catalog number: 9102, Cell Signaling), rabbit anti-β-actin (Catalog number: 8457, Cell Signaling), rabbit anti-p-TAK1 (Ser412; Catalog number: 9339, Cell Signaling), rabbit anti-TAK1 (Catalog number: 5206, Cell Signaling), mouse anti-TLR4 (Catalog number: sc-293072, Santa Cruz Biotechnology), rabbit anti-MyD88 (Catalog number: 4283, Cell Signaling), mouse anti-TRAF6 (Catalog number: sc-8409, Santa Cruz Biotechnology), fluorescein isothiocyanate (FITC)-conjugated goat anti-rabbit IgG (Catalog number: A11034, Invitrogen, Carlsbad, CA, USA), and horseradish peroxidase-conjugated anti-rabbit (Catalog number: 111-035-003) or anti-mouse IgG (Catalog number: 115-035-003, Jackson ImmunoResearch Laboratories, West Baltimore Pike, PA, USA). 

### 4.2. Microglial Cell Culture

A c-myc-immortalized mouse microglial cell line, namely, MG6 (RIKEN Cell Bank, RCB2403, Tsukuba, Japan), was established from the primary cultured microglia as described in previous study [48]. The MG6 cells retain many of the phenotypic features of primary microglial cells in terms of the morphological characteristics, phagocytic function, microglial-specific molecular expression, and proinflammatory cytokine responses to LPS [48,49]. MG6 cells were routinely maintained in a growth medium composed of Dulbecco’s modified Eagle’s medium (DMEM) containing 10% fetal bovine serum (FBS), 100 μg/mL streptomycin, and 100 U/mL penicillin in 100 mm Petri dishes (BD Falcon). The cells were never cultivated beyond passage 20, and the medium was changed every 2 days. MG6 cells (2.6 × 104 cells/cm^2^) were seeded onto glass coverslips or into six-well plates. 

### 4.3. Hp-s1 Ganglioside Administration

The cells were transferred to serum-free DMEM before the pharmacological treatment was initiated. They were pretreated with Hp-s1 for 2 h before the treatment with LPS (Catalog number: L4391, Sigma, St. Louis, MO, USA). Hp-s1 was kindly provided by Dr. Yow-Fu Tsai (Chung Yuan Christian University, Taoyuan, Taiwan), who synthesized Hp-s1 as described previously [22,50]. 

### 4.4. Cell viability Assays

Cell viability was measured with a Cell Counting Kit-8 (CCK-8) assay (Dojindo, Kumamoto, Japan). Briefly, 10 μL of the aliquot of the CCK-8 solution was added to each well of a 24-well plate; the cells were then incubated at 37 °C for another 2 h, and absorbance was measured at 450 nm by using a microplate reader.

### 4.5. Immunofluorescence

The cells on the coverslips were fixed in 10% neutral buffered formalin for 10 min, washed with phosphate-buffered saline (PBS), permeabilized, and blocked with 0.5% non-fat milk and 0.2% Triton X-100 for 15 min. The primary antibody was then added and incubated overnight. The cells were washed with PBS thrice for 5 min, and the secondary antibody (FITC-conjugated rabbit or mouse anti-goat IgG) was added and incubated at room temperature for 1 h. The cells were washed again with PBS. Then, coverslips were mounted with 3% n-propyl gallate and 50% glycerol in PBS, and the specimens were viewed under a fluorescence microscope (Nikon, Tokyo, Japan). The nuclei were labeled with DAPI (4′,6-diamidino-2-phenylindole, 1 μg/mL; Catalog number: 40043, Biotium Inc., Fremont, CA, USA) for 10 min. 

### 4.6. Western Blot Analysis

The cells were homogenized in a radioimmunoprecipitation assay (RIPA) buffer lysis buffer (50 mM Tris, pH 7.4, 150 mM NaCl, 0.1% sodium dodecyl sulfate (SDS), 1% Nonidet P-40, 1 mM ethylenediaminetetraacetic acid (EDTA), and 0.5% sodium deoxycholate) containing 1 mM phenylmethylsulfonyl fluoride (PMSF), 2 mM Na_3_VO_4_, 5 mM sodium fluoride (NaF), and a protease inhibitor cocktail. The protein concentration in the supernatant was quantified with a BCA kit (Thermo Scientific, Rockford, IL, USA) in accordance with the manufacturer’s instructions. Afterward, 30 μg of the protein was loaded onto 10–15% SDS-polyacrylamide gel electrophoresis (SDS–PAGE) gels. After electrophoresis, the proteins were transferred to nitrocellulose membranes. The membranes were blocked with a 5% blocking solution (5% non-fat milk in Tris-buffered saline [TBS] with 0.1% Tween 20 [TBS-T]) for 1 h at room temperature, incubated with primary antibodies at 4 °C overnight, and further incubated with horseradish peroxidase (HRP)-conjugated secondary antibodies (1:5000) for 1 h. Then, the membranes were washed with TBS-T and incubated with enhanced chemiluminescence reagents (ECL; Thermo Scientific) to visualize the bands. Each experiment was repeated at least three times. Immunoreactive bands were subjected to densitometric analysis by using ImageJ software (Version 1.46r., NIH, Bethesda, MD, USA).

### 4.7. Statistics

Quantitative data were presented as the mean ± SD of at least three independent experiments. Data were statistically analyzed using an unpaired two-tailed t-test or one-way analysis of variance (ANOVA) followed by Bonferroni’s post hoc test to determine statistical significance. Differences were considered significant at * *p* < 0.05 and highly statistically significant at ** *p* < 0.01. 

## 5. Conclusions

In conclusion, the current study first demonstrated that biological function of the Hp-s1 ganglioside against neuroinflammation. Hp-s1 inhibited LPS-induced iNOS, COX-2, TNF-α, IL-6, and IL-1β expressions by downregulating the expression of MyD88 and TRAF6, thereby blocking the activation of NF-κB and JNK/p38 MAPK in MG6 microglial cells (Figure 7). Neuroinflammation is a common pathogenesis of many neurological disorders. The experimental findings support that Hp-s1 might be developed as a novel compound for treating neuroinflammation-mediated diseases involving activated microglial cells.
